# Impact of two rounds of mass drug administration using diethylcarbamazine combined with albendazole on the prevalence of *Brugia timori *and of intestinal helminths on Alor Island, Indonesia

**DOI:** 10.1186/1475-2883-4-5

**Published:** 2005-07-13

**Authors:** Tim Oqueka, Taniawati Supali, Is Suhariah Ismid, Purnomo, Paul Rückert, Mark Bradley, Peter Fischer

**Affiliations:** 1 Bernhard Nocht Institute for Tropical Medicine, Bernhard-Nocht-Strasse 74, D-20359 Hamburg, Germany; 2 Department of Parasitology, Faculty of Medicine, University of Indonesia, Salemba 6, Jakarta 10430, Indonesia; 3 U.S. Naval Medical Research Unit No. 2, Jakarta, Indonesia; 4 German Technical Co-operation (GTZ), P.O. box 1217, Kupang 85000, Indonesia; 5 Global Community Partnerships, GlaxoSmithKline, 980 Great West Road, Brentfort Middlesex TW8 9GS, U.K; 6 Department of Internal Medicine, Infectious Diseases Division, Washington University School of Medicine, 660 S. Euclid, Campus box 8051, St. Louis, MO 63110, USA

## Abstract

**Background:**

Annual mass drug administration (MDA) using diethylcarbamizine (DEC, 6 mg/kg) combined with albendazole (alb, 400 mg) is recommended by the Global Programme to Eliminate Lymphatic Filariasis (GPELF). This strategy has been shown to be efficient in the of control bancroftian filariasis, but data on brugian filariasis as well as on the positive side effects on intestinal helminths are lacking.

**Methods:**

The effect of one selective treatment and two rounds of MDA using DEC and alb on the prevalence and intensity of *Brugia timori *infection were studied on Alor island using a cross-sectional and a cohort approach. Before the campaign and ten months after each treatment cycle microfilariae (mf) were assessed by filtration of night blood. Before and ten months after MDA, stool samples were collected and the prevalence of intestinal helminths were determined.

**Results:**

In all, the mf-rate dropped from 26.8% before any treatment to 3.8% following the second MDA. Almost all mf-positive, treated individuals showed very low mf densities. The crude prevalence of hookworm dropped from 25.3% to 5.9%. The reduction of prevalence of *Ascaris lumbricoides *(32.3% to 27.6%) and *Trichuris trichiura *(9.4% to 8.9%) was less pronounced. Within a cohort of 226 individuals, which was examined annually, the prevalence of *A. lumbricoides *dropped from 43.8% to 26.5% and of *T. trichiura *from 12.8% to 6.6%. The results indicate that this MDA approach reduces not only the mf prevalence of *B. timori *but also the prevalence of hookworm and to a lesser extent also of *A. lumbricoides *and *T. trichiura*.

**Conclusion:**

The MDA using DEC and alb as recommended by GPELF is extremely effective for areas with brugian filariasis. The beneficial effect of MDA on intestinal helminths may strengthen the national programme to eliminate lymphatic filariasis in Indonesia and may set resources free which are otherwise used for deworming campaigns of schoolchildren.

## Background

Lymphatic filariasis (LF) has been targeted by the World Health Organization for elimination as a public health problem by the year 2020 [1,2]. It is caused by three species of filarial parasites: *Wuchereria bancrofti *infects about 115 million people in Africa, India and other tropical and subtropical areas, whereas *Brugia malayi *infects about 13 million people in south India and south-east Asia and it is replaced by its sibling species *Brugia timori *in eastern Indonesia and Timor-Leste [3]. In Asia, the key strategy of the Global Programme to Eliminate LF (GPELF) is an annual mass drug adminstration (MDA) of all individuals at risk of infection with a single annual dose of diethylcarbamazine (DEC) combined with albendazole (alb) for at least four to five subsequent years [2,4]. This approach has been shown to reduce microfilaraemia of *W. bancrofti *and *B. malayi *efficiently [5-7]. Studies in areas endemic for *W. bancrofti *also indicate that the treatment with DEC combined with alb has the additional long-term beneficial effect of reducing prevalence and intensity of infection with intestinal helminths such as *Ascaris lumbricoides*, hookworms and *Trichuris trichiura *[8-11].

In 2001, The Department of Health of the Indonesian Government decided to participate in the GPELF. Although Indonesia has a long history in filariasis control programmes [12], filariasis is still in many areas a large public health problem and the new strategy recommended by GPELF was never evaluated. Therefore, studies were initiated to investigate the efficacy of a single dose DEC combined with alb to control *W. bancrofti *and *B. timori *infections on Alor island [13]. The treatment was judged to be efficient and safe enough to be employed in an MDA approach and an area endemic for *B. timori *was selected for annual follow-up studies [13-15].

In the present study we examined the prevalence of *B. timori *microfilaraemia following one selective treatment and two annual rounds of MDA using DEC combined with alb in a highland village of Alor island. We assessed the prevalence of intestinal helminths before MDA and its impact on the prevalence of the most common intestinal helminths, *A. lumbricoides*, hookworms and *T. trichiura*. We provide data on the effectiveness of two annual single doses of DEC and alb treatment to control *B. timori *and intestinal helminths.

## Methods

### Study area

The study was performed in Mainang village on Alor island (East Nusa Tenggara Timor, Indonesia). This village was first characterized in April 2001, and it was found to have a prevalence of *B. timori *microfilaraemia of about 25%. [16]. About 40% of the inhabitants showed signs of infection and 80% presented IgG4 antibodies reactive with a recombinant *B. malayi *antigen, BmR1 [16,17]. *Anopheles barbirostris *mosquitoes were identified as vectors for *B. timori *[18]. An animal reservoir for this *Brugia *species is not known and wild-life as well as domestic animals were rare in the study village. Before treatment intestinal helminths were common, especially *A. lumbricoides *(prevalence 2002, 32.3%) and hookworms (prevalence 2002, 25.3%) as well as *T. trichuria *(prevalence 2002, 9.4%). In the study area a few individuals received diethylcarbamazine (DEC) before 1990 and no anthelminthics were commercially available on the island. However, a deworming campaign was performed in schoolchildren at irregular intervals using generic benzimidazole derivates before the year 2000. In the village about 60% of eligible children attended school.

### Sample collection

Originally, the study was planned as a cohort-study with annual re-examinations. Since the most appropriate travel period to Alor is also harvesting season, many farmers were absent from their home during the re-examinations. Therefore, in 2001, 2002, 2003 and 2004 between 37 and 51% of the total resident population (about 1,500 individuals) were examined and data were analysed using a cross-sectional approach. The percentage of newly registered individuals, decreased from 45% during the first annual re-examination in 2002 to 25% in 2003 and to 20% in 2004. By summarizing all surveys, about 90% of the resident population living in the area in 2001 attended at least one survey. Only 145 (10%, 70 males, 75 females, median age in the year 2001, 26 years) individuals participated in all four filariasis surveys and 226 (15%, 117 males, 109 females, median age in the year 2002, 22 years) in the three surveys which included stool collection. Data of these individuals were also analysed separately as a cohort.

In May 2001 about 200 selected individuals with microfilariae (mf) and/or lymphoedema received a single dose of DEC (6 mg/kg) combined with alb (400 mg) [13]. This number represented about 40% of the total number of individuals with mf and/or lymphoedema in the study area. A second survey was performed in April/May 2002 to assess the long-term efficacy of the filariasis treatment in selected individuals. The treatment results of these mf-positive individuals were reported previously [13,14]. For comparison their data were also included in this report. In addition, during the 2002 survey, baseline data on the prevalence of intestinal helminths were collected. Re-examination following MDA was carried out from April to May 2003 and from March to April 2004. Inhabitants of the endemic villages were called by their local health workers to a central place, usually the Puskesmas (primary health center). For each volunteer, sex, age and name were noted and after a brief clinical examination venous blood was collected between 7.00 p.m. and 11 p.m. One labeled stool container was provided to each individual participating in the study and collected one day later by local health workers.

The participants came from three different residential quarters of Mainang, Welai Selatan, Malaipea and Tominuku, but no significant differences with regard to the prevalence of *B. timori *or intestinal helminths were found [16]. All individuals over the age of two years were asked to participate in the study. Informed consent was obtained from all adults or, in the case of children, from their parents. The study was approved by the ethical board of the University of Indonesia, Jakarta. Following the registration, the individuals were examined by experienced physicians for clinical signs of lymphatic filariasis. During the survey patients with lymphoedma were introduced to hygiene of affected legs and other procedures which may help to stop the progression or alleviate symptoms of their disease.

### Mass drug administration

In May and June 2002 and 2003, after each survey, a community based MDA using a single dose of DEC and alb for the entire eligible population was performed. Pregnant or breastfeeding women, children younger than two years or persons suffering from acute illness were not treated. A medication regimen based on the classification of age rather than on weight was used (Table 1), since this was found to facilitate treatment.

**Table 1:** Simplified dosing regimen used for mass drug administration of DEC (100 mg tablets) and albendazole (400 mg tablets) to control lymphatic filariasis on Alor island, Indonesia, compared to the average body weight (2002, standard deviation, SD).

Age	DEC	Albendazole	Mean body weight in kg (SD)
2–6 years, pre-school	1 tablet	1 tablet	14.4 (6.4)
7–12 years, primary school	2 tablets	1 tablet	23.6 (5.2)
13 years and older, high school and adults	3 tablets	1 tablet	45.7 (9.1)

The MDA of the rest of the island was conducted by the District Health Authority, which claims a coverage rate (number of distributed doses per number of residents) of 78%. Staff from the GTZ performed MDA in 6 randomly selected *B. timori *and/or *W. bancrofti *endemic villages with a total population of about 6,000 inhabitants and introduced a developed educational programme [19]. For MDA a mixed approach was used, involving both, the primary health care system and the community members directly. Health staff and volunteers were trained for correct drug distribution and documentation as well as side effect recognition and its treatment. The impact of this campaign was tested by using KAP (knowledge, attitudes, practice) surveys before and after the intervention [19]. In this project a MDA coverage rate of 75% was achieved, whereas 15% of the population, according to WHO guidelines, were not eligible for treatment. Besides the effect of treatment the educational programme has increased the knowledge about filariasis. In our study village Mainang in April 2003 a compliance rate (number of persons who reported to have taken the drug per number of residents) for the 2002 MDA of 67% was determined (n = 772).

### Assessment of microfilariae

For the identification of mf-positive individuals, 1 ml of anticoagulated blood (EDTA) was filtered through a polycarbonate membrane with a 5 μm pore size (Millipore, Eschborn, Germany). Subsequently mineral water was passed through the membrane for blood cell lysis. The membrane was placed on a slide, air-dried and fixed with methanol. Following Giemsa staining, slides were air-dried, examined microscopically using 100-fold magnification and mf were counted.

### Assessment of helminth eggs

In the field, the Harada-Mori hatching test was used to detect living hookworm larvae. Briefly about 0.5 g fresh stool was spread on a wet filter paper and placed with 1 ml water in a specially designed plastic bag. The bags were placed upright in a window, covered by paper and incubated for 6 to 10 days. The samples were examined microscopically for the presence of hookworm larvae at a 63-fold magnification. The rest of the stool samples were preserved in the field using 4% formaldehyde. In the laboratory in Jakarta, 1–2 g of stool was examined by the formalin/ether enrichment method for the presence of helminth eggs. The most prevalent geohelminths, *A. lumbricoides*, hookworm and *T. trichiura *were analysed in this study, since other species such as *Hymenolepis spp*. and *Strongyloides stercoralis *were only found in a few cases.

In the baseline survey in 2002 the Kato-Katz smear was used to assess the helminth eggs quantitatively. Compared to the enrichment method and the Harada-Mori test the Kato Katz technique had a poor sensitivity and this method was not applied in the following years. In 2002 the number of eggs per gram (epg) in infected individuals as determined by the Kato Katz smear was usually relatively low with medians for *A. lumbricoides*, hookworms and *T. trichiura *of 3,500 epg, 50 epg and 75 epg, respectively. For the enrichment method and the Harada Mori test, the numbers of helminth eggs or hookworm larvae were scored as follows: low density (1–10 eggs per slide or 1–50 hookworm larvae per plastic bag), moderate density (11–100 eggs or 51–500 larvae) or high density (more than 100 eggs or 500 larvae). Usually, scoring data are in good agreement with results obtained by the Kato Katz Smear [20].

### Statistical analysis

*EpiInfo 2002 Revision2 *was used for documentation and analysis of the data. As index for the mf density within a study group the geometric mean was used. For the estimation of the community mf load (CMFL) a log (x+1) transformation was used. Data on distribution and density of mf were compared using the chi-square test or the Mann-Whitney U test.

## Results

### Brugia timori

**Figure 1:**
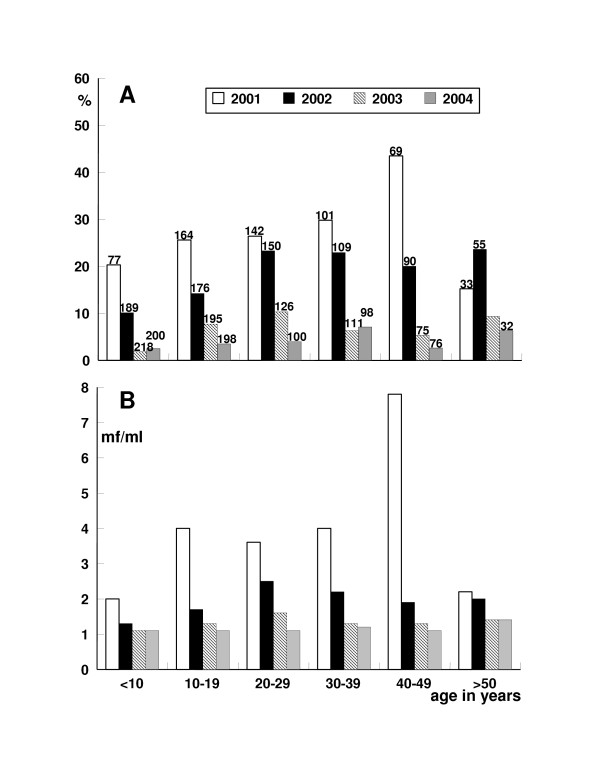
(A) Prevalence of *B. timori *mf positive individuals in Mainang village, Alor, Indonesia, from 2001 to 2004 by age. The number of individuals examined is noted on top of each column. Selective DEC/albendazole treatment was performed after the survey in 2001. MDA was performed after the surveys in 2002 and 2003. (B) Communtiy microfilarial load (CMFL) of *B. timori *in Mainang village, Alor, Indonesia, from 2001 to 2004 by age. Due to definition the minimum of CMFL is 1 microfilaria per ml night blood (mf/ml).

In the years 2001 to 2004 a total of 586, 769, 768, and 704 individuals, respectively, were examined for the presence of mf (Table 2, Fig. 1). In 2001, a *B. timori *mf prevalence of 26.8% was observed, which dropped one year after selective treatment in 2002 to 17.6 %. In 2003, about one year following the first round of MDA, a mf prevalence of 6.1% was detected, while in 2004, about one year following the second round of MDA, a mf prevalence of 3.8% was recorded (Fig. 1A). There was no difference in prevalence reduction between male and female individuals (P > 0.05). In total, from 2001 to 2004 a reduction of mf prevalence of 85% was observed.

**Table 2:** Number of individuals examined for *B. timori *from 2001 to 2004 grouped by mf density and the numbers of individuals who claimed to have been treated with DEC/albendazole in the previous year.

Mf/ml	2001 No. (%)		2002 No. (%)		2003 No. (%)		2004 No. (%)	
	
	Examined*	Treated**	Examined*	Treated**	Examined*	Treated**	Examined*	Treated**
0	429 (73.2)	0	634 (82.4)	126 (19.9)	721 (93.9)	482 (66.9)	677 (96.2)	548 (80.9)
1–100	66 (11.3)	0	90 (11.7)	22 (24.4)	25 (3.3)	17 (68.0)	20 (2.8)	14 (70.0)
101–500	42 (7.2)	0	29 (3.8)	6 (20.7)	14 (1.8)	6 (42.9)	4 (0.6)	2 (50.0)
>500	49 (8.3)	0	16 (2.1)	1 (6.3)	8 (1.0)	4 (50.0)	3 (0.4)	0 (0)
Total	586 (100)	0	769 (100)	155 (20.5)	768 (100)	509 (66.3)	704 (100)	564 (80.1)

*percentage of total examined, **percentage of negative or positive examined

The CMFL, the geometric mean number of mf per examined person including the mf-negative individuals, dropped from 3.8 mf/ml in 2001 to 1.1 mf/ml in 2004 (Fig. 1B). However, due to the used definition of the CMFL its minimum is 1 mf/ml and in areas with low prevalence and low mf densities the CMFL becomes inaccurate. While in 2001, 31.2% of 157 mf-positive individuals had high mf densities of more than 500 mf/ml, this percentage decreased in 2002 to 12.2% of 135, in 2003 to 17.0% of 47 and in 2004 to 11.1% of 27 microfilaraemics (Table 2).

The mf-positive individuals, who received their first treatment in 2001, were re-examined after six, twelve, 24 and 34 months. In 2004, 73 of these individuals could be re-examined. Most of them had received three treatments and the prevalence dropped from 100% to 5.5%. The geometric mean mf density of the mf-positive individuals dropped from 142 mf/ml before treatment (2001) to 1.1 mf/ml after 34 months (2004).

**Figure 2:**
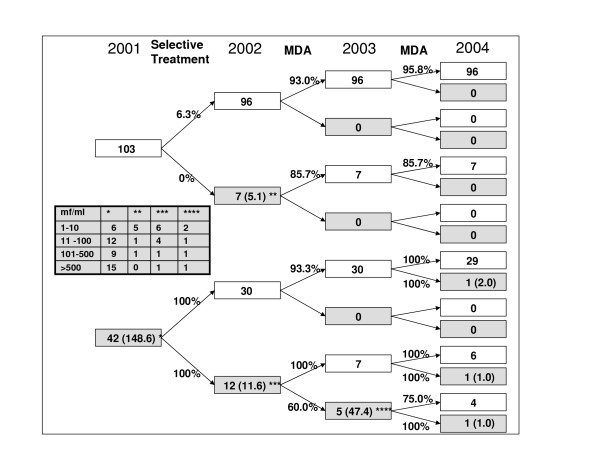
Number of people examined each year from 2001 to 2004 for *B. timori *and geometric mean of the mf-positives. The white boxes are mf-negative individuals, the grey boxes the mf-positive individuals. The percentages on the arrow indicate the treatment compliance. In the table the boxes marked with the stars differentiate the mf density.

In order to determine the dynamics of the mf-status during the investigation period, the mf prevalence in a cohort of 145 individuals, who participated in all four annual surveys, was determined (Fig. 2). In this group 42 (29.0%) individuals, with a geometric mean mf density of 148.6 mf/ml, were mf-positive in 2001. In 2002, 7 formerly mf-negative individuals became microfilaraemic, while 30 formerly mf-positive individuals became amicrofilaraemic after the selective DEC and alb treatment. In 2003, following the first round of MDA, no new microfilaraemics were observed, while 5 individuals remained mf-positive. Three of these individuals had very low mf densities. Two individuals claimed not to have participated in MDA a year before. In both individuals the mf density increased from 2002 to 2003 from 5 to 27 mf/ml and from 726 to 2,723 mf/ml, respectively. In 2004, following the second round of MDA two individuals became microfilaraemic who were mf-negative the year before and one person remained microfilaraemic. The mf density for the 3 mf-positives, who were all positive at the beginning of the observation period, was 1.3 mf/ml, which is a reduction from 2001 to 2004 of >99% for the mf-positives (Fig. 2).

### Ascaris lumbricoides

**Figure 3:**
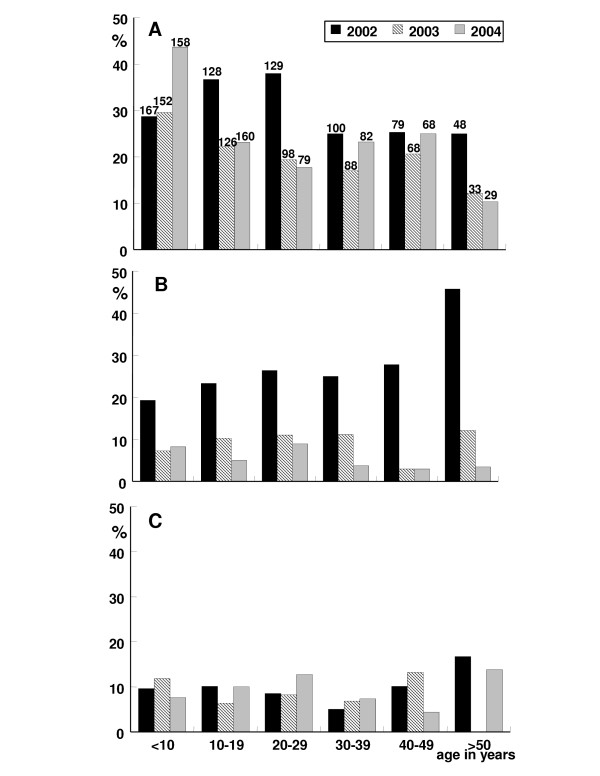
(A) Prevalence of *A. lumbricoides *positive individuals in Mainang village, Alor, Indonesia, from 2002 to 2004 by age. The number of persons examined is noted on top of each column. DEC/albendazole MDA was performed after the surveys in 2002 and 2003. (B) Prevalence of hookworm positive individuals in Mainang village, Alor, Indonesia, from 2002 to 2004 by age. (C) Prevalence of *T. trichuria *positive individuals in Mainang village, Alor, Indonesia, from 2002 to 2004 by age.

To asses the effects of the MDA on the prevalence of intestinal helminths stool samples were collected in 2002 before MDA and in 2003 and 2004 about 10 months after MDA. In the years 2002, 2003 and 2004 stool samples were collected from 651, 565 and 576 individuals, respectively (Table 3, Fig. 3).

**Table 3:** Number of individuals examined for intestinal helminths from 2002 to 2004 grouped by infection status with *A. lumbricoides*, hookworms and *Trichuris trichiura *and the numbers of individuals who claimed to have been treated with DEC/albendazole in the previous year.

	2002 No. (%)		2003 No. (%)		2004 No. (%)	
	
	Examined*	Treated**	Examined*	Treated**	Examined*	Treated**
*Ascaris*						
Negative	450 (67.8)	84 (18.7)	440 (77.9)	309 (70.2)	417 (72.4)	357 (85.6)
Positive	201 (32.2)	26 (12.9)	125 (22.1)	98 (78.4)	159 (27.6)	121 (76.1)

Hookworm						
Negative	486 (74.7)	90 (18.5)	515 (91.8)	376 (73.0)	542 (94.1)	458 (84.5)
Positive	165 (25.3)	20 (12.1)	50 (8.2)	31 (62.0)	34 (5.9)	20 (58.8)

*Trichuris*						
Negative	590 (90.6)	104 (17.6)	516 (91.3)	369 (71.5)	525 (91.1)	435 (82.9)
Positive	61 (9.4)	6 (6.6)	49 (8.7)	38 (77.6)	51 (8.9)	43 (84.3)

Total	651 (100)	110 (16.9)	565 (100)	407 (72.0)	576 (100)	478 (83.0)

*percentage of total examined, **percentage of negative or positive examined

**Figure 4:**
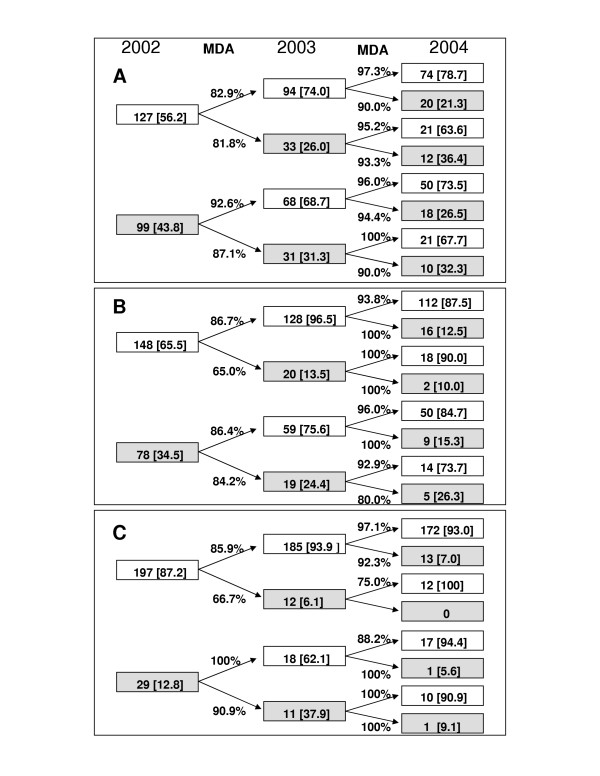
(A) Number of people examined each year from 2002 to 2004 for *Ascaris *and the percentage (in brackets) of the infected (egg-positive) and non-infected (egg-negative) individuals for each group. The percentages on the arrow indicate the treatment compliance. (B) Number of people examined each year from 2002 to 2004 for hookworm and the percentage of the infected and non-infected individuals for each group. The percentages on the arrow indicate the treatment compliance. (C) Number of people examined each year from 2002 to 2004 for *Trichuris *and the percentage of the infected and non-infected individuals for each group. The percentages on the arrow indicate the treatment compliance.

The crude prevalence of *A. lumbricoides *dropped from 32.3% in 2002 to 22.1% in 2003. However, in 2004 a crude prevalence of 27.6% was observed (Table 3). No difference in prevalence reduction was observed in male and female individuals. Children under the age of ten years had the highest infection rate of 28.7% (2002), 29.6% (2003) and 43.7% (2004), whereas in the other age groups no increase of prevalence was observed (Fig. 3A). In contrast to these crude data, a significant decrease of *A. lumbricoides *prevalence was observed in a cohort of 226 individuals, who were examined in 2002, 2003 and 2004 (Fig. 4A). In these three years the prevalence dropped in this cohort from 43.8% to 28.3% and to 26.5%. This is a reduction of 39.7% after two rounds of MDA with a compliance rate of 86% and 95%. In 2003, 33 new infections occurred, while 68 individuals who were infected with *A. lumbricoides *became negative. In 2004, 38 new infections occurred, while 41 persons turned negative (Fig. 4A). In 2003 and 2004 about 90% of the infected individuals had light infections with an estimate of less than 2,000 eggs /g stool, whereas before treatment 50% of the examined individuals had 3,500 eggs /g or more.

### Hookworms

The crude prevalence of hookworm infection decreased from 25.3% in 2002 to 8.2% in 2003 and the 5.9% in 2004 (Table 3). This is an average reduction of prevalence of 76.7%. In children the rate of infection dropped from 19.3 to 7.2% in 2003 and remained low with 8.2% in 2004, while in the other age groups the decrease of prevalence continued also in 2004 (Fig. 3B). No difference in hookworm reduction was observed in male and female individuals. In the cohort of 226 individuals with full annual data sets, the prevalence dropped from 2002 to 2004 from 34.5% to 17.3% and 14.2%, respectively. This equals following two rounds of MDA within this time course to a reduction of 59% (Fig. 4B). In 2003, 20 new infections were observed and 59 individuals became hookworm negative. In 2004, 25 new infections were detected, while 32 persons turned negative. This corresponds to an average rate of new infections of 13.5% and an average clearance rate of about 80%. The mean egg count before MDA was already relatively low, with about 65% of individuals with light infections of less than 2,000 eggs /g. In 2003 and 2004 the intensity of infection was even lower with more than 90% having light infections. The results from the formol/ether enrichment methods were in principal confirmed by the data obtained from Harada Mori culture, although in a few cases the hatching test was more sensitive.

### Trichuris trichiura

The crude prevalence of *T. trichiura *was almost stable from 2002 to 2004 with only a non-significant decrease from 9.4% to 8.7% and to 8.9%, respectively (Table 3). The prevalence of infected children under the age ten years increased only slightly from 9.6% in 2002 to 11.8% in 2003, but dropped to 7.6% in 2004 (Fig. 3C). A more pronounced reduction of prevalence was observed in the cohort of 226 individuals, who where examined in all three years. In this group the prevalence was in 2002, 12.8% and a year later it was 10.2% and in 2004 it was only 6.6%. This is a significant reduction over two years of 48.4%. In 2003, 18 individuals became negative for *T. trichiura *eggs, while 12 new infections occurred. A year later 22 individuals turned negative, while 2004, 14 new infections occurred, of which 13 individuals were negative at the surveys in 2002 and 2003 (Fig. 4C). The rate of new infections in 2003 and 2004 was 6.1% and 7.0% respectively. In this cohort 80% of the individuals who were positive in 2002 became negative for *T. trichiura *in 2004, but new infections occurred. The mean egg count before MDA was already very low, with about 80% of individuals having light infections of less than 2,000 eggs /g. In 2003 and 2004 the intensity of infection continued to be low and almost all individuals had light infections.

## Discussion

This study shows the effect of one selective treatment and two rounds of MDA using DEC combined with alb on the most prevalent helminths, *B. timori*, *A. lumbricoides*, hookworms and *T. trichiura *in a community on Alor island. The results confirm and show for the first time at the community level, that the MDA approach as recommended by the GPELF is highly effective at reducing the prevalence and the intensity of infection of *B. timori*. Furthermore, a positive impact on the prevalence, especially of hookworm infection, but also of *A. lumbricoides *and of *T. trichiura*, was observed. The data support the hypothesis that *B. timori *is an excellent candidate for elimination, while the campaign will also have a large impact on the reduction of intestinal helminths [15]. *B. timori *occurs only east of the Wallace line in Indonesia and Timor-Leste. This restricted distribution makes this species not only a good candidate for local elimination, but *B. timori *may also be a prime candidate for eradication of lymphatic filariae [15,21].

*B. timori *is closely related to *B. malayi *and tools and strategies developed to support the elimination of *B. malayi *infection may apply for both species [13-15,17,18]. It has been shown that the combination of a single annual dose of DEC combined with alb is very efficient in the control of brugian filariasis and a higher efficacy of this regimen has been suggested as compared to bancroftian filariasis [6,13,14]. The successful control of *B. timori *on parts of Flores island using multiple doses of DEC has been reported already some decades ago [12]. However, this strategy has caused many logistical problems and was never extended to the remaining parts of Flores and other islands. The present study proved the principle that a single annual dose of DEC combined with alb is highly suitable to control and most probably to eliminate *B. timori *infection. Before treatment, among a cohort of 145 individuals, 45 were mf-positive with a geometric mean mf density of almost 150 mf/ml. Three years later, following one selective treatment and two rounds of MDA in this group only 3 individuals were found to be mf-positive, who had mf densities of 1–2 mf/ml.

Our data show that a larger percentage of mf-positive individuals claimed not to have participated in MDA, compared to the average compliance rate. It can be concluded that a high compliance rate is necessary for reducing the mf prevalence to levels under which transmission cannot be sustained. In addition, a few individuals who received no treatment still had high mf densities. If these individuals participate in the next rounds of MDA, side effects may occur and affected individuals may spread their problems in the community which eventually reduce compliance. However, extensive health information campaigns can help to ensure high compliance rates [19].

On the community level not much data exists about the control of brugian filariasis by annual MDA using DEC combined with alb. For the control of *W. bancrofti *infection in Asia a number of extensive field studies were published using annual MDA with DEC alone or in combination with alb. Results from Papua New Guinea indicate promising prospects for elimination [22,23]. In India, after six rounds of a single dose treatment with DEC the prevalence of microfilaraemics was reduced by 86% and the mf density by 91% [7]. Computer models predict that in this area a further decline of mf prevalence will occur even after the cessation of MDA [24]. In this study *(B.timori)*, similar reductions of prevalence and mf density were observed after only one selective treatment and two rounds of MDA. It is possible that models would also predict a further decline of mf prevalence for our study, but epidemiological parameters differ largely between *W. bancrofti *infections in Pondicherry (India) and *B. timori *infections on Alor island and further studies on the dynamics of *B. timori *control are needed. In addition, it has been discussed for *W. bancrofti *in the pacific area that MDA should be accompanied by local vector control [25].

Our results showed that MDA using a combination of DEC and alb also has an impact on the reduction of intestinal nematode infections. In the highland village examined the original prevalence and intensity of infections with *A. lumbricoides*, hookworms and *T. trichiura *was relatively low. This is in agreement with previous surveys on Alor and on other islands of volcanic origin in east Nusa Tenggara Timur [26,27]. Although, both hookworm species occur in eastern Indonesia, data from Flores indicate that *Necator americanus*, may be the prominent species [27].

The strongest reduction of prevalence following two rounds of MDA among the intestinal helminths was observed in hookworm infections, in both, the cross-sectional group of an average of 600 individuals and a cohort of 226 individuals. Although a large number of re-infections occurred, the crude prevalence dropped from 25.3% to 5.9% and in the cohort from 34.5% to 14.2%, ten months after the second round of MDA. This equals a reduction of 76.7% and 58.8%, respectively. The hookworm prevalence is usually reduced by about 80% shortly after treatment with alb [28,29]. Although DEC alone may reduce the output of hookworm eggs, it is assumed that it has no influence on its prevalence [30]. In our study we observed a large number of hookworm re- or new infections. In 13.5% (2003) and 14.0% (2004) of the cohort new infections were observed. From Java, an even higher re-infection rate with *N. americanus *of about 50% one year after anthelminthic treatment was reported [31]. Despite of the occurrence of re-and new infections, the drop in hookworm prevalence can be explained by the relatively short survival time of hookworm larvae in the environment as compared to the mean survival time of eggs of *A. lumbricoides *and of *T. trichiura*.

Following the first round of MDA the crude prevalence of *A. lumbricoides *dropped from 32.2% to 22.1%, but after the second round it was 27.6%. More consistently were the results in the cohort. Before MDA the prevalence was 43.3%, following the first round it was 28.3% and following the second round it was 26.5%. Albendazole is very effective against *A. lumbricoides *and median cure rates are over 95% [28,29,32]. DEC alone has a minor therapeutic effect on *Ascaris *[10,33]. Although some treated individuals may expel adult worms after DEC, the overall prevalence of infection may be not affected [30]. These observations are confirmed by other studies, which show that DEC alone has no significant impact on *A. lumbricoides*, but the combination of DEC with alb has relevant cure and egg reduction rates [34]. As in other intestinal helminths, re- and new infections occur regularly, and the time point of re-examination is critical. In our cohort we observed an annual rate of new infections of 25%. Children have an especially high risk for re- or new-infections and show a lower decrease in worm burden compared to adults [35]. In another study it was observed that eight months after treatment 55% of children were re-infected [36]. Re-infection with *A. lumbricoides *may return six months after treatment to almost 90% of the pre-treatment prevalence and worm density may drop to about 75% [35].

The crude prevalence of *T. trichiura *in the community before and after MDA was almost identical, ranging between 9.4% and 8.7%. However, following two rounds of MDA the prevalence dropped in the cohort from 12.8% to 6.6%. Although re- and new infections occurred, it is important to note that in 2004 most new infections were observed in those individuals which were negative for *T. trichiura *for the previous two years. *Trichuris trichiura *is known to be only poorly sensitive to albendazole and the reported reduction rates for alb range between 38% and 47.7% [28,29,32]. From Sri Lanka a cure rate of *T. trichiura *of 43.6% and an egg reduction rate of 70.3% was reported [37]. The combination of DEC with alb showed different results, ranging from no significant impact on the prevalence but with significant egg reduction of 79.4% one week after treatment, to a cure rate of 81.6% and an egg reduction of 84% [10,34]. The study from Sri Lanka reported that this drug combination has a cure rate of 30% and an egg reduction rate of 70% [36].

For areas endemic for *W. bancrofti *there are an increasing number of studies which show the positive effect of filariasis control using MDA with DEC combined with alb on the reduction of intestinal helminths [8-11,38]. The results of the present study can extend this observation to areas endemic for *Brugia *infections. Although it is unlikely that MDA as used for filariasis elimination will eliminate intestinal helminths from most areas, a reduction may be achieved to levels which may cause no significant morbidity. Other intervention strategies, such as for example the development of a hookworm vaccine [39], may take advantage of reduced prevalences in order to achieve a long-lasting elimination of intestinal helminths, as it has been accomplished in most industrialised countries. In areas with filariasis control by the MDA using DEC combined with alb, separate de-worming campaigns for school-age children may become superfluous. This could set available resources free which can then be used to support MDA. Co-ordination is needed within the local health administration to use the limited funds more efficiently. The present study showed that MDA using DEC combined with alb is effective to control *B. timori *and that this has also impact on the reduction of geohelminths.

## Conclusion

Annual MDA using DEC in combination with alb is highly effective in the control of *B. timori *infection and has a positive impact on the reduction of intestinal helminths. Given a high compliance rate the strategy can lead to elimination of *B. timori *on Alor and on other islands in Indonesia.

## List of abbreviations

Alb, albendazole

CMFL, community microfilarial load

DEC, diethylcarbamazine

GPELF, Global Programme to Eliminate Lymphatic Filariasis

MDA, mass drug admistratrion

Mf, microfilariae

## Competing interests

Mark Bradley is employee of GlaxoSmithKline, which donate albendazole for filariasis elimination.

## Authors' contributions

Tim Oqueka cand. MD, participated in field work, performed data analysis and wrote the first draft of the manuscript

Taniawati Supali, PhD, conceived the study, participated in field work, performed stool examinations and edited the manuscript

Is Suhariah Ismid MD, participated in the base-line survey and made comments on the manuscript

Purnomo PhD, participated in the base-line survey, performed stool examinations and made comments on the manuscript

Paul Rückert MD, MPH, PhD conceived the study, provided logistic support and edited the manuscript

Mark Bradley PhD, conceived the study, helped with data analysis and with writing of the manuscript

Peter Fischer PhD, conceived the study, participated in all field surveys, helped with data analysis and drafted the manuscript

## References

[B1] Bebehani K (1998). Candidate parasitic diseases. Bull World Health Organ.

[B2] Ottesen EA, Duke BO, Karam M, Behbehani K (1997). Strategies and tools for the control/elimination of lymphatic filariasis. Bull World Health Organ.

[B3] Michael E, Bundy DA (1997). Global mapping of lymphatic filariasis. Parasitol Today.

[B4] Molyneux D (2003). Lymphatic filariasis (elephantiasis) elimination: a public health success and development opportunity. Filaria J.

[B5] Horton J, Witt C, Ottesen EA, Lazdins JK, Addiss DG, Awadzi K, Beach MJ, Belizario VY, Dunyo SK, Espinel M, Gyapong JO, Hossain M, Ismail MM, Jayakody RL, Lammie PJ, Makunde W, Richard-Lenoble D, Selve B, Shenoy RK, Simonsen PE, Wamae CN, Weerasooriya MV (2000). An analysis of the safety of the single dose, two drug regimens used in programmes to eliminate lymphatic filariasis. Parasitology.

[B6] Shenoy RK, John A, Babu BS, Suma TK, Kumaraswami V (2000). Two-year follow-up of the microfilaraemia of asymptomatic brugian filariasis, after treatment with two, annual, single doses of ivermectin, diethylcarbamazine and albendazole, in various combinations. Ann Trop Med Parasitol.

[B7] Ramaiah KD, Vanamail P, Pani SP, Yuvaraj J, Das PK (2002). The effect of six rounds of single dose mass treatment with diethylcarbamazine or ivermectin on *Wuchereria bancrofti *infection and its implications for lymphatic filariasis elimination. Trop Med Int Health.

[B8] De Silva NR, Pathmeswaran A, Fernando SD, Weerasinghe CR, Padmasiri EA, Montressor A (2003). Impact of mass chemotherapy for the control of filariasis on geohelminth infections in Sri Lanka. Ann Trop Med Parasitol.

[B9] Rajendran R, Mani TR, Munirathinam A, Sunish IP, Abdullah SM, Augustin DJ, Satyanarayana K (2003). Sustainability of soil-transmitted helminth control following a single-dose co-administration of albendazole and diethylcarbamazine. Trans R Soc Trop Med Hyg.

[B10] Mani TR, Rajendran R, Munirathinam A, Sunish IP, Abdullah S, Augustin DJ, Satyanarayana K (2002). Efficacy of co-administration of albendazole and diethylcarbamazine against geohelminthiases: a study from South India. Trop Med Int Health.

[B11] Mani TR, Rajendran R, Sunish IP, Munirathinam A, Arunachalam N, Satyanarayana K, Dash AP (2004). Effectiveness of two annual, single-dose mass drug administrations of diethylcarbamazine alone or in combination with albendazole on soil-transmitted helminthiasis in filariasis elimination programme. Trop Med Int Health.

[B12] Partono F, Maizels RM, Purnomo (1989). Towards a filariasis-free community: evaluation of filariasis control over an eleven year period in Flores, Indonesia. Trans R Soc Trop Med Hyg.

[B13] Supali T, Ismid I, Rückert P, Fischer P (2002). Treatment of *Brugia timori *and *Wuchereria bancrofti *infections in Indonesia using DEC or a combination of DEC and albendazole: adverse reactions and short-term effects on microfilariae. Trop Med Int Health.

[B14] Fischer P, Djuardi Y, Ismid I, Rückert P, Bradley B, Supali T (2003). Long-lasting reduction of *Brugia timori *microfilariae following a single dose of diethylcarbamazine combined with albendazole. Trans R Soc Trop Med Hyg.

[B15] Fischer P, Supali T, Maizels RM (2004). Lymphatic filariasis and *Brugia timori*: prospects for elimination. Trends Parasitol.

[B16] Supali T, Wibowo H, Rückert P, Fischer K, Ismid I, Purnomo, Djuardi Y, Fischer P (2002). High prevalence of *Brugia timori *infection in the highland of Alor island, Indonesia. Am J Trop Med Hyg.

[B17] Supali T, Rahmah N, Djuardi Y, Sartono E, Ruckert P, Fischer P (2004). Detection of filaria specific IgG4 antibodies using Brugia Rapid test in individuals from an area highly endemic for *Brugia timori*. Acta Trop.

[B18] Fischer P, Wibowo H, Pischke S, Ruckert P, Liebau E, Ismid IS, Supali T (2002). PCR-based detection and identification of the filarial parasite *Brugia timori *from Alor Island, Indonesia. Ann Trop Med Parasitol.

[B19] Krentel A, Rückert P, Servais G, Manoempil P, Fischer P A knowledge, attitudes and practice (KAP) survey of lymphatic filariasis to prepare and evaluate mass drug administration in Alor District, Indonesia.

[B20] Muller R (2002). Worms and Human Disease.

[B21] Molyneux DH, Hopkins DR, Zagaria N (2004). Disease eradication, elimination and control: the need for accurate and consistent usage. Trends Parasitol.

[B22] Bockarie MJ, Tisch DJ, Kastens W, Alexander ND, Dimber Z, Bockarie F, Ibam E, Alpers MP, Kazura JW (2002). Mass treatment to eliminate filariasis in Papua New Guinea. N Engl J Med.

[B23] Bockarie MJ, Kazura KW (2003). Lymphatic filariasis in Papua New Guinea: prospects for elimination. Med Microbiol Immunol.

[B24] Subramanian S, Stolk WA, Ramaiah KD, Plaisier AP, Krishnamoorthy K, Van Oortmarssen GJ, Dominic Amalraj D, Habbema JD, Das PK (2004). The dynamics of *Wuchereria bancrofti *infection: a model-based analysis of longitudinal data from Pondicherry, India. Parasitology.

[B25] Burkot T, Ichimori K (2002). The PacELF programme: will mass drug administration be enough?. Trends Parasitol.

[B26] Joesoef A, Dennis DT (1980). Intestinal and blood parasites of man on Alor Island Southeast Indonesia. Southeast Asian J Trop Med Public Health.

[B27] Higgins DA, Jenkins DJ, Kurniawan L, Purnomo, Harun S, Juwono SS (1984). Human intestinal parasitism in three areas of Indonesia: a survey. Ann Trop Med Parasitol.

[B28] Horton J (2000). Albendazole: a review of anthelminthic efficacy and safety in humans. Parasitlogy.

[B29] Bennett A, Guyatt H (2000). Reducing intestinal nematode infection: efficacy of albendazole and mebendazole. Parasitol Today.

[B30] Meyrowitsch DW, Simonsen PE (2001). Short communication: efficacy of DEC against *Ascaris *and hookworm infections in schoolchildren. Trop Med Int Health.

[B31] Soeripto N (1991). Reinfection and infection rates of soil-transmitted-helminths in Kemiri Sewu, Yogyakarta, Indonesia. Southeast Asian J Trop Med Public Health.

[B32] Ottesen EA, Ismail MM, Horton J (1999). The role of albendazole in programmes to eliminate lymphatic filariasis. Parasitol Today.

[B33] Turner P, Michael E (1997). Recent advances in the control of lymphatic filariasis. Parasitol Today.

[B34] Belizario VY, Amarillo ME, de Leon WU, de los Reyes AE, Bugayong MG, Macatangay BJC (2003). A comparison of the efficacy of single doses of albendazole, ivermectin and diethylcarbamazine alone or in combinations against *Ascaris *and *Trichuris *spp. Bull World Health Organ.

[B35] Hall A, Anwar KS, Tomkins AM (1992). Intensity of reinfection with *Ascaris lumbricoides *and its implications for parasite control. Lancet.

[B36] Hagel I, Lynch NR, Di Prisco MC, Perez M, Sanchez JE, Pereyra BN, Soto de Sanabria I (1999). Helminth infection and anthropometric indicators in children from a tropical slum: *Ascaris *reinfection after anthelminthic treatment. J Trop Pediatr.

[B37] Ismail MM, Jayakody RL (1999). Efficacy of albendazole and its combinations with ivermectin or diethylcarbamazine (DEC) in the treatment of *Trichuris trichiura *infections in Sri Lanka. Ann Trop Med Parasitol.

[B38] Pani SP, Subramanyam Reddy G, Das LK, Vanamail P, Hoti SL, Ramesh J, Das PK (2002). Tolerability and efficacy of single dose albendazole, diethylcarbamazine citrate(DEC) or co-administration of albendazole with DEC in the clearance of *Wuchereria bancrofti *in asymptomatic microfilaraemic volunteers in Pondicherry, South India: a hospital-based study. Filaria J.

[B39] Hotez PJ, Zhan B, Bethony JM, Loukas A, Williamson A, Goud GN, Hawdon JM, Dobardzic A, Dobardzic R, Ghosh K, Bottazzi ME, Mendez S, Zook B, Wang Y, Liu S, Essiet-Gibson I, Chung-Debose S, Xiao S, Knox D, Meagher M, Inan M, Correa-Oliveira R, Vilk P, Shepherd HR, Brandt W, Russell PK (2003). Progress in the development of a recombinant vaccine for human hookworm disease: the human hookworm vaccine initiative. Int J Parasitol.

